# Combating echinococcosis in China: strengthening the research and development

**DOI:** 10.1186/s40249-017-0374-3

**Published:** 2017-11-21

**Authors:** Men-Bao Qian, Bernadette Abela-Ridder, Wei-Ping Wu, Xiao-Nong Zhou

**Affiliations:** 1 National Institute of Parasitic Diseases, Chinese Center for Disease Control and Prevention, Shanghai, 200025 China; 2 Key Laboratory of Parasite and Vector Biology, Ministry of Health, Shanghai, 200025 China; 3 National Center for International Research on Tropical Diseases, Shanghai, 200025 China; 4 World Health Organization Collaborating Center for Tropical Diseases, Shanghai, 200025 China; 5 Department of Control of Neglected Tropical Diseases, World Health Organization, 1211 Geneva, Switzerland

**Keywords:** Echinococcosis, Research and development, Health China 2030, Belt and Road Initiative

## Abstract

Echinococcosis is a neglected zoonotic disease, causing great morbidity and mortality due to the wide distribution of its endemic areas. China holds a high percentage in the global burden of both cystic and alveolar echinococcosis. A national survey conducted between 2012 and 2016 showed that an estimated 50 million people are at risk of contracting the disease in western China, of whom about 0.17 million are cases with echinococcosis.

Despite this, research and development on echinococcosis in China is greatly inadequate compared to that in other countries. In this paper, we argue that there is a need for more research and work to be conducted in China on echinococcosis, including researching techniques in regards to diagnosis, treatment, and vaccination, and developing products through technical transformation and piloting strategies to control and even elimination.

However, great opportunities exist for China to strengthen the research and development on this disease through initiatives such as Health China 2030, the Belt and Road Initiative, the China-Africa cooperation, as well as through further cooperation between China and the World Health Organization. All of these can bring us closer to controlling echinococcosis in China as well as in other countries. One element of crucial importance will be the training and development of professionals, which can be strengthened through international cooperation.

**Electronic supplementary material:**

The online version of this article (10.1186/s40249-017-0374-3) contains supplementary material, which is available to authorized users.

## Multilingual abstracts

Please see Additional file 1 for translations of the abstract into the five official working languages of the United Nations.

## Introduction

Echinococcosis is a zoonotic tropical disease caused by adult or larval stages of *Echinococcus* spp. The two major species of medical and public health importance are *E. granulosus* and *E. multilocularis*, which cause cystic echinococcosis and alveolar echinococcosis, respectively [1].

Echinococcosis leads to significant morbidity and mortality, especially alveolar echinococcosis, with a high fatality rate if not managed in a timely manner. Cystic echinococcosis has a global distribution in most pastoral and rangeland areas, with high endemicity in the eastern part of the Mediterranean, northern Africa, southern and eastern Europe, southern part of South America, Central Asia, Siberia, and western China [2]. Alveolar echinococcosis has a narrow distribution in the northern hemisphere, especially in China, the Russian Federation, and countries in continental Europe and North America [2].

The great burden caused by echinococcosis not only includes human health being affected or human lives lost due to significant morbidity and mortality, but also the economic loss relating to livestock husbandry [3]. The latter also worsens the former by weakening accessibility to medical intervention.

In this paper, we argue that as a country with a high burden of both cystic and alveolar echinococcosis, China should strengthen research on and development of products, strategies, and professionals related to echinococcosis. This will promote the control and eventually the elimination of echinococcosis in China, as well as in other endemic areas.

## Current situation of echinococcosis in China

### High burden of echinococcosis in China

Both cystic echinococcosis and alveolar echinococcosis are highly endemic in western China [2].

It was estimated that a total of one million disability-adjusted life years (DALYs) were caused by cystic echinococcosis globally, out of which 0.40 million were in China [3]. Additionally, out of the US$ 1.92 billion lost annually that was attributed to cystic echinococcosis globally, China was responsible for US$ 0.66 billion [3]. The annual global livestock production losses associated with cystic echinococcosis were also high, reaching US$ 2.19 billion, of which China held an important share [3].

Approximately 18 235 new alveolar echinococcosis cases were estimated to occur each year globally, out of which 16 629 (91%) were in China [4]. The global DALYs due to alveolar echinococcosis were 666 433 and the corresponding years of life lost (YLLs) were 616 897, of which 95% were in China [4]. A national survey conducted on echinococcosis in China between 2012 and 2016 demonstrated that a population of about 50 million in nine western provinces are at risk of contracting and nearly 170 000 people are cases with echinococcosis [5].

At least 368 counties are endemic with cystic echinococcosis in China, out of which 119 are co-endemic with alveolar echinococcosis [5]. Although the percentage of burden in the globe shared by China is not as high as early estimations [3, 4], according to the aforementioned national survey [5], China is still the most important endemic area for both cystic and alveolar echinococcosis.

### Echinococcosis in China: The state of research and development

Research and development pertaining to a certain disease determines its control potential. The capacity of research and development is partially reflected through scientific publications, especially those accessible by international societies, namely in the English language.

**Fig. 1:**
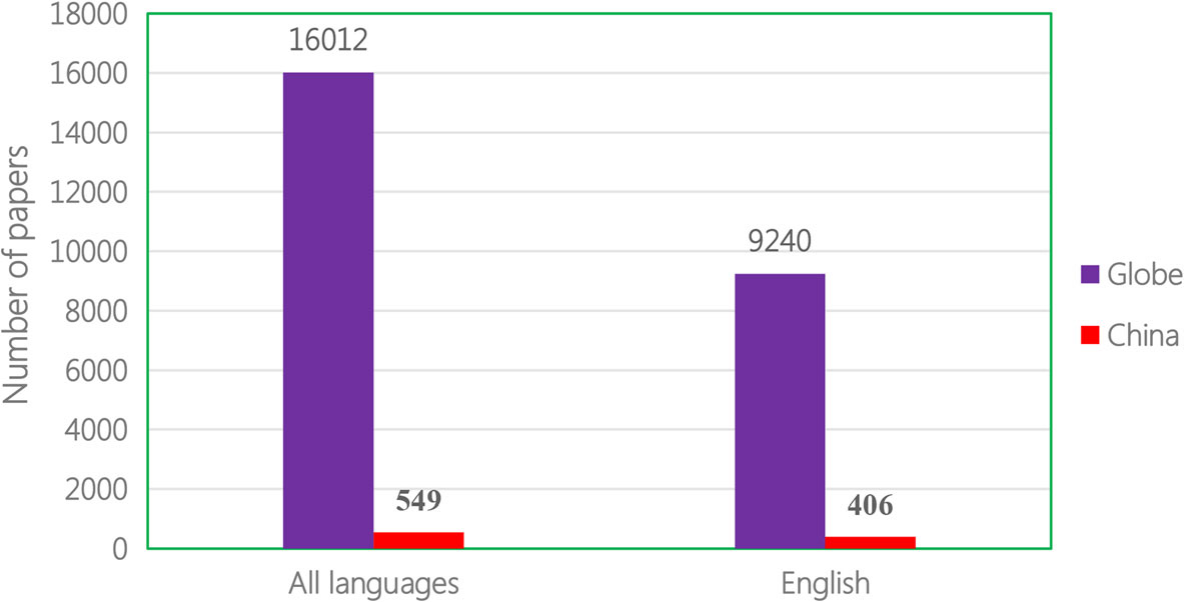
The number of scientific papers on echinococcosis documented in PubMed

**Fig. 2:**
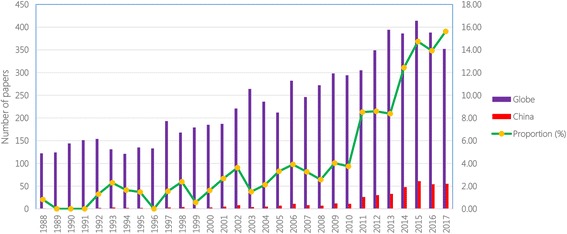
Changing trend of the number of scientific papers in the English language on echinococcosis documented in PubMed

After searching the most important scientific database, namely PubMed (see Additional file 2), the following gaps in echinococcosis research and development in China were found: 16 012 documented papers on echinococcosis exist in PubMed, including 9 240 in English (see Fig. 1), however, of those only 549 and 406, respectively, were contributed by Chinese scientists. This clearly demonstrates that although there is a high burden of echinococcosis in China, the output in research and development (namely scientific reports) is inadequate in the country. This indirectly demonstrates the weakness of research and development on echinococcosis in China. Fortunately, this disadvantageous situation is changing, which is demonstrated through the gradually increasing proportion in published papers by Chinese scientists (see Fig. 2).

## The roadmap to strengthening research on and development of products, strategies, and professionals related to echinococcosis in China

**Fig. 3:**
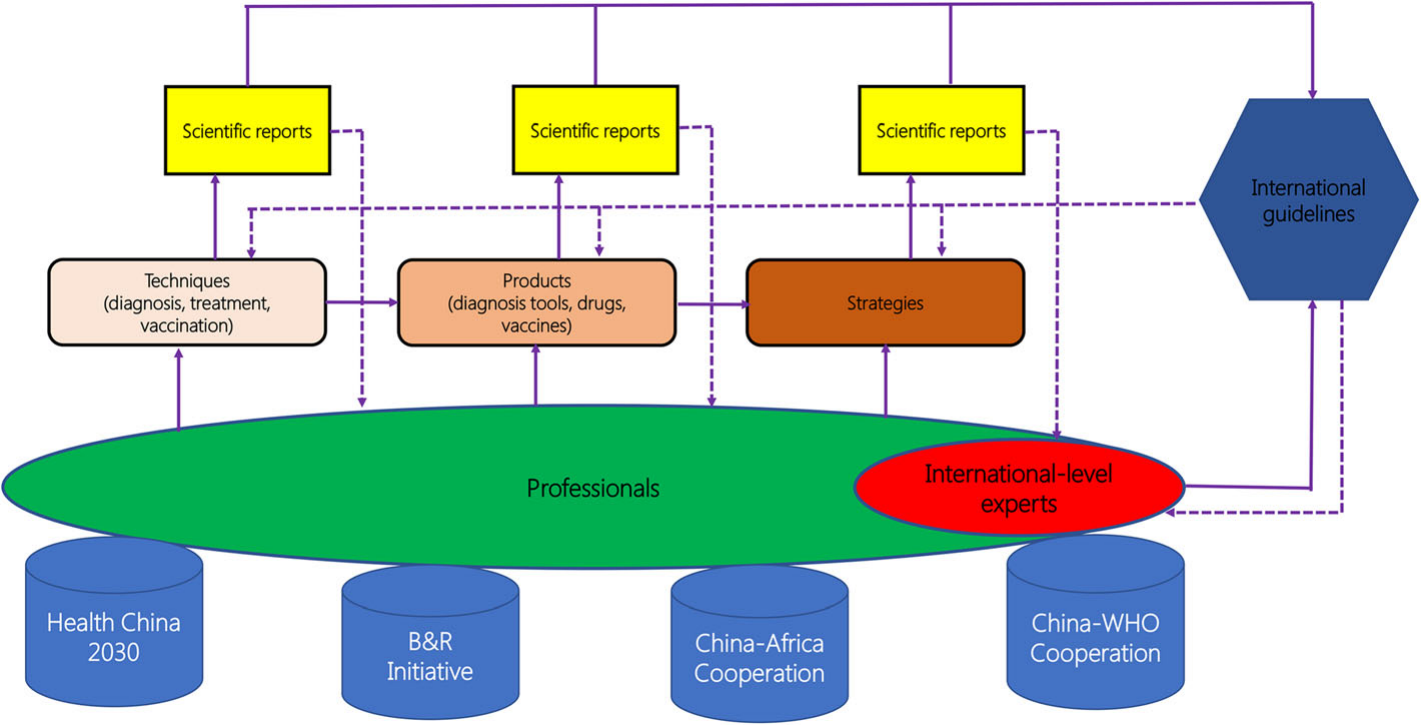
A schematic diagram showing the approaches to strengthen research and development on echinococcosis in China (solid arrows represent forward role and dashed arrows represent reverse role)

Fig. 3 shows a schematic diagram outlining how to strengthen the capacity in research and development on echinococcosis in China. The framework involves various, often interlaced, levels, including research on techniques and their development, personnel training, and use of available platforms, as well as international cooperation and participation.

### Research and development

Research and development comprises researching techniques, developing products and piloting strategies.

Gaps in the techniques required to control echinococcosis in China include intervention tools such as diagnosis, treatment, and vaccines for both humans and animals due to the zoonotic characteristics of the disease [6]. For example, field-friendly and validated serological tests for humans with high sensitivity and specificity are currently not available. Due to the high co-endemicity of cystic and alveolar echinococcosis in China [5], it is valuable but also challenging to develop the immunological diagnosis tools for humans that could differentiate the species. Furthermore, cross-reactivity with other zoonotic cestodes, namely *Taenia* spp., is also a concern as the latter is also co-endemic in western China [7].

Currently, echinococcosis is treated with benzimidazoles (albendazole or mebendazole), but the long treatment duration and side effects are challenging the compliance of patients. This requires either the development of a short-term effective drug for treatment and/or the optimization of the current treatment strategy.

Regarding definitive hosts, such as dogs, frequent deworming (e.g. monthly dosing) is challenging in terms of coverage and sustainability due to the wide spatial scope of endemic areas in western China and the associated need for an enormous input of resources. Thus, a validated slow-release drug is required, which could positively influence the blocking of transmission sources [8]. Furthermore, new techniques to deliver baits (e.g. the delivery by unmanned aerial vehicles) in stray dogs and wild canines are also highly valuable, which will save the man-power and promote the coverage of deworming [9]. Especially, it will also partially alleviate the challenges in the control of transmission sources of alveolar echinococcosis. The EG95 vaccine is being given to sheep in China [10], but a vaccine targeting dogs has not yet been successful. The development of this vaccine will not only contribute to the control of cystic echinococcosis, but also alveolar echinococcosis.

The development of products transformed from innovative techniques is also very important. Only when techniques are transformed into products and validated in the field can the research be considered meaningful. The development of products should follow international standards, which will promote uptake in other endemic countries.

Although there are several successful examples of controlling and even eliminating cystic echinococcosis in the world, the progress is very slow [10]. Different environments in western China are not totally comparable, and other countries that have tackled echinococcosis did not necessarily experience the same severity of the disease as China has/is. In terms of alveolar echinococcosis, there have hardly been any examples of successful control from which China could learn [10]. Thus, control strategies should be piloted in China, including international and domestic-made techniques and products. In addition, more explorations on the social, ecological, and cultural determinants should be supported, which will guide the formulation of effective control strategies. The strategies should vary according to the endemic types (mere cystic echinococcosis, co-endemicity of cystic and alveolar echinococcosis) and the endemic areas.

Furthermore, during research and development, the outputs of scientific reports are also important, as they promote the exchange of knowledge, techniques, products, and concepts, and can eventually be used to establish international guidelines. Importantly, scientific reports should be written in English, as these are the ones that are accessible to international societies.

### Development and training of professionals and importance of international participation

Owing to the overall success in the control of neglected tropical diseases, human resources spent on these diseases in China are shrinking [11]. Fostering professional teams is of utmost importance in supporting research and work on echinococcosis in the country. These professional teams should include people from clinical, laboratory, and field disciplines. Within these teams, the development of experts at the international level will be crucial. As well as scientific outputs, international-level experts are another important channel to export the achievements made in research and development. Furthermore, international-level experts could also introduce and incorporate new techniques, products, and concepts from international societies into China’s control strategies.

International participation will be important both in the fostering of general talent and international-level experts. International participation will promote their development, which means that more opportunities will be provided, especially for young professionals, through international cooperation.

### Available platforms

Despite the disease’s ranking among neglected tropical diseases globally, more and more opportunities are becoming available to combat echinococcosis in China.

In October 2016, China issued the “Healthy China 2030 Planning Outline”, putting health at the center of the country’s entire policy-making. The outline specifies that echinococcosis should be under control at the county level by 2030 [12].

The Belt and Road Initiative has also progressed successfully, and the project encompasses nearly two-thirds of the world’s total population [13]. Health is also recognized as one important domain of this initiative, including the necessity of cooperation to combat major infectious diseases [14]. The initiative connects Asia with Europe and Africa, areas that encompass the foci of echinococcosis. Therefore, under the framework of the Belt and Road Initiative, the cooperation potential is great for combating echinococcosis.

In December 2015, the Johannesburg Summit and the 6th Ministerial Conference of the Forum on China-Africa Cooperation were held in Johannesburg [15]. According to the sequential action plan, China should assist Africa to develop public health systems and policies; help African countries to improve public health, surveillance, epidemiological, and prevention systems; and strengthen prevention and treatment of malaria and other common infectious and communicable diseases in Africa. Echinococcosis, as one important neglected tropical disease in Africa [2], should also benefit from this initiative.

In January 2017, China’s president Xi Jinping, visited the World Health Organization (WHO), which was the first time a Chinese president visited the WHO [16]. In August 2017, the WHO Director-General Tedros Adhanom Ghebreyesus visited China [17]. China and the WHO are aligned in their vision and have strengthened their partnership, including through a mutual commitment to the Belt and Road Initiative. The “universal health coverage” proposed by the WHO inherently requires research and development on and subsequent control of echinococcosis [18].

## Conclusions

Echinococcosis, as one important zoonotic tropical disease in China, afflicts people living in western endemic areas. However, gaps exist pertaining to research and development required to support the control of the disease. There are also, however, great opportunities for research and development owing to the many available platforms. Just like the control and elimination of other neglected tropical diseases in China, the control of echinococcosis will not only contribute to better health of Chinese people, but also promote control of the disease in other endemic countries through the exportation of techniques, products, and professionals.

## Additional files

Additional file 1: Multilingual abstracts in the five official working languages of the United Nations. (PDF 447 kb)

Additional file 2: The methodology in search of the number of papers documented in PubMed on echinococcosis. (DOCX 19 kb)

## Abbreviations

DALYs: Disability-adjusted life years
US$: US dollar
WHO: World Health Organization
YLLs: Years of life lost
