# Supplementary material for: Combating echinococcosis in China: strengthening the research and development
Source: Infect Dis Poverty. 2017 Nov 21;6:161. doi: 10.1186/s40249-017-0374-3 (PMC5697071; doi:10.1186/s40249-017-0374-3)
Supplement: Supplementary file 1 — Multilingual abstracts in the five official working languages of the United Nations. (PDF 447 kb) [file 40249_2017_374_MOESM1_ESM.pdf]

Translation of the abstract into the six official working languages of the United Nations

تعزيز البحوث والتطورات في ما يتعلّق بمرض المُشوِّكات في الصين

الرجال-باو تشيان، برناديت أبيلا ريدر، وي بينغ وو، شياو نونغ زو

ملخص
إنّ مرض المُشوِّكات (إشينوكوكوسيس) هو مرض مهمل ذا منشأ حيواني، يتسبب بنسبة عدوى هائلة وعدد وفيات ضخم في المناطق الموبوءة وعلى نطاق واسع. وتشارك الصين بنسبة عالية من العبء العالمي لكل من مرضي المُشوِّكات الكيسي والسنخي. وقد اظهر استطلاع وطنى حديث ان هناك حوالى 50 مليون شخص معرضون لخطر الإصابة بهذا المرض فى غرب الصين، منهم حوالى 0.17 مليون مصاب بمرض المُشوِّكات. ومع ذلك، فإن البحوث حول مرض المُشوِّكات في الصين غير كافية إلى حد بعيد مقارنةً مع تلك الحاصلة في بلدان أخرى. لقد تناقشنا من أجل زيادة البحث والتطوير لمواجهة مرض المُشوِّكات في الصين بما في ذلك البحث عن التقنيات وتطوير المنتجات وواضعي الاستراتيجيات. وسيكون تدريب أصحاب المواهب ذا أهمية جوهريّة من خلال المشاركة الدولية. وهناك الآن فرص كبيرة من خلال مبادرة الصين للصحة 2030 ومبادرة الحزام والطريق والتعاون بين الصين وأفريقيا، فضلا عن المزيد من التعاون بين الصين ومنظمة الصحة العالمية، الأمر الذي يمكن أن يجعلنا قريبين من السيطرة على مرض المُشوِّكات في الصين، وكذلك الأمر في بلدان أخرى.

Translated from English version into Arabic by Tameem Harb, through

TRANSLATORS
WITHOUT BORDERS

加强中国包虫病的研发

Men-Bao Qian, Bernadette Abela-Ridder, Wei-Ping Wu, Xiao-Nong Zhou

摘要
包虫病是一种被忽视的人兽共患病，在广大的流行区造成明显的发病和死亡。中国在全球囊型包虫病和泡型包虫病疾病负担中占据了很大的比重。最新的全国调查显示中国西部地区有 5000 万人受到包虫病威胁，其中 17 万患有包虫病。但与其他国家相比，中国在包虫病的研发明显不足。因此，我们认为中国需要加强包虫病研发工作，包括新技术的研究和产品的开发以及策略的试点。而通过国际化合作来培养人才是重要支撑。“健康中国 2030”、“一带一路”战略、中非合作以及不断深化的中国和世界卫生组织之间的合作都是重要的机遇，这些机遇使中国和全球更加接近包虫病控制的目标。

Translated from English version into Chinese by Men-Bao Qian

Intensifier la recherche et les programmes de développement sur l'échinococcose en Chine

Men-Bao Qian, Bernadette Abela-Ridder, Wei-Ping Wu, Xiao-Nong Zhou

Résumé
L'échinococcose est une maladie zoonotique négligée, avec des chiffres de morbidité et de mortalité

élevés dans les régions d'endémie à haut risque. La Chine posséde une part élevée en pourcentage dans la charge mondiale de l'échinococcose cystique et alvéolaire. Une enquête nationale actualisée a révélé qu'environ 50 millions de personnes sont exposées au risque de développer la maladie dans l'ouest de la Chine, quelque 0,17 million d'entre elles sont atteintes d'échinococcose. Cependant, la recherche sur l'échinococcose en Chine est bien trop insuffisante par rapport à celle menée dans d'autres pays. Nous avons défendu la nécessité d'effectuer davantage de recherche et d'études de développement sur l'échinococcose en Chine, incluant celles portant sur les techniques, les découvertes de produits et sur les projets pilotes en matière de stratégies. Les talents et la formation seront d'une importance capitale à travers la participation internationale. De grandes perspectives existent aujourd'hui grâce au plan " La Chine en bonne santé 2030", au projet "La ceinture et la route", à la coopération Chine-Afrique ainsi qu'au renforcement de la coopération entre la Chine et l'OMS, nous permettant de faire avancer la lutte contre l'échinococcose en Chine, ainsi que dans d'autres pays.

Translated from English version into French by veromarie, through

TRANSLATORS
WITHOUT BORDERS

Укреплять научные исследования и разработки по эхинококкозу в Китае

Цянь Мэнь-Бао, Бернадетт Абела-Риддер, У Вэй-Пин, Чжоу Сяо-Нун

Аннотация

Эхинококкоз - это забытая зоонозная болезнь, приводящая к высокой заболеваемости и смертности в широко эндемичных районах. Доля Китая в общей заболеваемости кистозным и альвеолярным эхинококкозом является очень большой. Обновленные данные национального обследования показали, что в Западном Китае примерно 50 миллионов человек подвергаются риску заболевания, из которых около 0,17 миллиона заражены эхинококкозом. Однако в Китае проводится слишком мало исследований эхинококкоза по сравнению с другими странами. Мы доказывали необходимость выполнения большего числа научных исследований и разработок в Китае по эхинококкозу, в том числе исследования по методам, разработка продуктов и пробных стратегий. Обучение специалистов при международном участии будет иметь решающее значение. В настоящее время для этого имеются большие возможности через программу "Здоровый Китай-2030", проект "Один пояс - один путь", китайско-африканское сотрудничество, а также дальнейшее взаимодействие между Китаем и ВОЗ, что может помочь нам в борьбе с эхинококкозом в Китае и других странах.

Translated from English version into Russian by Natalia Potashnik, through

TRANSLATORS
WITHOUT BORDERS

Fortalecer la investigación y el desarrollo de la equinococosis en China

Men-Bao Qian, Bernadette Abela-Ridder, Wei-Ping Wu, Xiao-Nong Zhou

Resumen

La equinococosis es una enfermedad zoonótica, que causa gran morbilidad y mortalidad en grandes áreas endémicas. China comparte un alto porcentaje en la carga mundial tanto de la equinococosis quística como la alveolar. Según muestra una actualizada encuesta nacional, se estima que unas 50 millones de personas están en peligro de contraer la enfermedad en China occidental, de las cuales aproximadamente 0,17 millones ya tienen equinococosis. Sin embargo, la investigación sobre la equinococosis en China es inadecuada comparada con la realizada en otros países. Pedimos más investigación y desarrollo sobre la equinococosis en China, -investigación sobre técnicas, desarrollo de productos y estrategias de proyectos piloto. La capacitación mediante la participación internacional será de crucial importancia. Ahora hay grandes oportunidades a través del programa China saludable 2030, la Iniciativa de Un cinturón, un camino, la cooperación entre China y África así también como mayor la cooperación entre China y la OMS. Todas pueden ayudarnos a controlar la equinococosis en China, así como en otros países.

Translated from English version into Spanish by cecidragonetti, through

TRANSLATORS
WITHOUT BORDERS
