# Supplementary material for: Combating echinococcosis in China: strengthening the research and development
Source: Infect Dis Poverty. 2017 Nov 21;6:161. doi: 10.1186/s40249-017-0374-3 (PMC5697071; doi:10.1186/s40249-017-0374-3)
Supplement: Supplementary file 2 — The methodology in search of the number of papers documented in PubMed on echinococcosis. (DOCX 19 kb) [file 40249_2017_374_MOESM2_ESM.docx]

**Supplement 1**

**The methodology in search of the number of papers documented in PubMed on echinococcosis**

**Database:**

PubMed: <https://www.ncbi.nlm.nih.gov/pubmed>

**Searching data:**

13 October 2017

**Searching strategy:**

Seeing **Table 1**.

**Searching results:**

Seeing **Table 1**.

**Table 1.** The searching strategy and results of the number of papers on echinococcosis in PubMed

| Search | Add to builder | Query | Items found | Time |
| --- | --- | --- | --- | --- |
| [#10](https://www.ncbi.nlm.nih.gov/pubmed/advanced) | [Add](https://www.ncbi.nlm.nih.gov/pubmed/advanced) | Search **(((((((Echinococcus[Title/Abstract]) OR Echinococcosis[Title/Abstract]) OR Hydatidosis[Title/Abstract]) OR Hydatid disease[Title/Abstract])) AND China[Affiliation])) AND English[Language]** | [406](https://www.ncbi.nlm.nih.gov/pubmed/?cmd=HistorySearch&querykey=10) | 08:43:37 |
| [#9](https://www.ncbi.nlm.nih.gov/pubmed/advanced) | [Add](https://www.ncbi.nlm.nih.gov/pubmed/advanced) | Search **(((((Echinococcus[Title/Abstract]) OR Echinococcosis[Title/Abstract]) OR Hydatidosis[Title/Abstract]) OR Hydatid disease[Title/Abstract])) AND China[Affiliation]** | [549](https://www.ncbi.nlm.nih.gov/pubmed/?cmd=HistorySearch&querykey=9) | 08:41:35 |
| [#8](https://www.ncbi.nlm.nih.gov/pubmed/advanced) | [Add](https://www.ncbi.nlm.nih.gov/pubmed/advanced) | Search **(((((Echinococcus[Title/Abstract]) OR Echinococcosis[Title/Abstract]) OR Hydatidosis[Title/Abstract]) OR Hydatid disease[Title/Abstract])) AND English[Language]** | [9240](https://www.ncbi.nlm.nih.gov/pubmed/?cmd=HistorySearch&querykey=8) | 08:40:47 |
| [#7](https://www.ncbi.nlm.nih.gov/pubmed/advanced) | [Add](https://www.ncbi.nlm.nih.gov/pubmed/advanced) | Search **China[Affiliation]** | [1093345](https://www.ncbi.nlm.nih.gov/pubmed/?cmd=HistorySearch&querykey=7) | 08:39:22 |
| [#6](https://www.ncbi.nlm.nih.gov/pubmed/advanced) | [Add](https://www.ncbi.nlm.nih.gov/pubmed/advanced) | Search **English[Language]** | [23096858](https://www.ncbi.nlm.nih.gov/pubmed/?cmd=HistorySearch&querykey=6) | 08:39:06 |
| [#5](https://www.ncbi.nlm.nih.gov/pubmed/advanced) | [Add](https://www.ncbi.nlm.nih.gov/pubmed/advanced) | Search **(((Echinococcus[Title/Abstract]) OR Echinococcosis[Title/Abstract]) OR Hydatidosis[Title/Abstract]) OR Hydatid disease[Title/Abstract]** | [16012](https://www.ncbi.nlm.nih.gov/pubmed/?cmd=HistorySearch&querykey=5) | 08:38:16 |
| [#4](https://www.ncbi.nlm.nih.gov/pubmed/advanced) | [Add](https://www.ncbi.nlm.nih.gov/pubmed/advanced) | Search **Hydatid disease[Title/Abstract]** | [3731](https://www.ncbi.nlm.nih.gov/pubmed/?cmd=HistorySearch&querykey=4) | 08:37:59 |
| [#3](https://www.ncbi.nlm.nih.gov/pubmed/advanced) | [Add](https://www.ncbi.nlm.nih.gov/pubmed/advanced) | Search **Hydatidosis[Title/Abstract]** | [2933](https://www.ncbi.nlm.nih.gov/pubmed/?cmd=HistorySearch&querykey=3) | 08:37:41 |
| [#2](https://www.ncbi.nlm.nih.gov/pubmed/advanced) | [Add](https://www.ncbi.nlm.nih.gov/pubmed/advanced) | Search **Echinococcosis[Title/Abstract]** | [8598](https://www.ncbi.nlm.nih.gov/pubmed/?cmd=HistorySearch&querykey=2) | 08:37:23 |
| [#1](https://www.ncbi.nlm.nih.gov/pubmed/advanced) | [Add](https://www.ncbi.nlm.nih.gov/pubmed/advanced) | Search **Echinococcus[Title/Abstract]** | [5948](https://www.ncbi.nlm.nih.gov/pubmed/?cmd=HistorySearch&querykey=1) | 08:37:04 |

**Note:** To avoid too many less relevant papers, the restrict with Title/Abstract was used. Thus, some relevant papers were probably missed. However, that should not impact the conclusion.
